# Parental representations after preterm birth: a narrative review

**DOI:** 10.3389/fpsyg.2023.1114418

**Published:** 2023-09-04

**Authors:** Emeline Hamon, Béatrice Bourdin, Barbara Le Driant

**Affiliations:** ^1^Centre de Recherche en Psychologie: Cognition, Psychisme et Organisations (UR 7273), Université de Picardie Jules Verne, Amiens, France; ^2^FHU “1000 jours pour la santé” prendre soin avant de soigner, Université de Lille, Lille, France

**Keywords:** preterm birth, parental representations, early interactions, attachment, parental mentalization, parental reflective functioning

## Abstract

Preterm birth accounts for nearly 15 million births annually worldwide and constitutes a considerable risk factor for atypical development. This birth context is a source of stress for the parents and often leads to an early separation between their child and them. Research on the influence of the birth status on the infant’s attachment style has shown no systematic link between preterm birth and the development of insecure attachment in children born preterm. This has opened up research perspectives in understanding the role of environmental factors. A literature review was conducted to present an overview of the current findings on parental representations (PR), particularly maternal ones, and their role in the context of preterm birth. PR quality appears to be associated with specific dyadic interaction patterns, thus exposing vulnerability factors. Studies exploring PR have pointed out the importance of considering parental mental elaboration mechanisms and contextual moderators in supporting socio-emotional development among children born preterm. We discussed the challenges of investigating PR in the context of preterm birth for future studies and emphasized the need for research studies to be conducted according to a developmental and non-deterministic perspective. This narrative review also aimed to highlight the importance of family centered care interventions in the context of a public policy focused on the child’s “First 1,000 days” of life.

## Introduction

Defined by the World Health Organization as a live birth occurring before 37 completed gestational weeks (GW), preterm birth accounted for 13.4 million births annually worldwide in 2020, or approximately 10% of births in the world (World Health Organization, 2023). Prematurity is a real public health issue on a global scale (Ryan and Dogbey, 2015). It constitutes a risk factor for the developmental trajectory of children born preterm in several aspects (Pinto-Martin et al., 2004; Johnson and Marlow, 2011; Nosarti et al., 2012; Treyvaud et al., 2012; De Rose et al., 2013; Healy et al., 2013; Witt et al., 2014; Zmyj et al., 2017; Brydges et al., 2018; Burstein et al., 2021; Crump et al., 2021). Moreover, prematurity causes stress and anxiety for the parents (Hollywood and Hollywood, 2011; Zelkowitz et al., 2011; Neri et al., 2015) and depression symptoms reported by NICU mothers (Segre et al., 2014). Preterm birth may have long-lasting effects and lead to symptoms of Post-Traumatic Stress Disorder (PTSD) (Suttora et al., 2014; Zerach et al., 2015). This event often leads to an early separation between the child and his or her parents, and appears to be a context of vulnerability for parenthood (Ionio et al., 2016; Treherne et al., 2017; Gonçalves et al., 2020), as well as for the development of optimal early interactions (Korja et al., 2012; Ionio et al., 2017). Previous findings on parent-preterm infant interaction indicated that mothers of premature infants could encounter bonding difficulties and difficulties to interpret the infant’s cues. In addition, preterm infants themselves interact differently compared to infants born at term, exhibiting less alertness, difficult behavioral regulation and providing less well-defined cues to the caregiver (Provasi, 2019 for a review). Thus, the hypothesis of an increased risk of insecure attachment in preterm children has been suggested. However, conflicting results can be identified in the literature (Brisch et al., 2005; Fuertes et al., 2011; Korja et al., 2012; Abergel and Blicharski, 2013). A meta-analysis by Korja et al. (2012) on the attachment style of children born preterm has found that these children do not present more insecure attachment patterns (evaluated around one year of age) than children born full-term, in line with a recent review by Genet and Devouche (2022). Nevertheless, a high risk of perinatal complications and developmental delays in the child can contribute to the development of an insecure attachment style (Laganière et al., 2003; Brisch et al., 2005; Udry-Jørgensen et al., 2011; Korja et al., 2012; Zengin Akkus et al., 2021). Most studies have also failed to consider the role of environmental influences when studying the relationship between birth status and infant attachment style, as pointed out by Laganière et al. (2003). This has opened up research perspectives in understanding the particularities of parental perceptions and the role of parental representations after the child’s preterm birth.

Parental representations (PR) consist of parents’ subjective experiences of how they perceive their infant, their role as a parent, and their relationship (Benoit et al., 1997). PR thus refer to a set of internal constructions specific to the parental function, created from an “accumulation of interactive stories” (Demers et al., 2009, p.209). Prenatal representations of the unborn child are shaped in the parents’ mind during pregnancy (Ammaniti, 1991; Dayton et al., 2010; Lindstedt et al., 2021) and predict mother-infant attachment (Fonagy et al., 1991b). Parental mental representations in the postpartum period remain consistent with the ones assessed in the prenatal period (Vreeswijk et al., 2015) and will result from a “subtle and rich integration and interplay with the baby’s temperament” (Bruschweiler-Stern, 2001, p.23).

The integration of the child’s own characteristics into the parents’ internal representations depends on the parent’s ability to understand and attend to the child’s mental state (Slade, 2005). PR can be evaluated through the parents’ imagination, elaboration, understanding, and description of the child. These parent’s abilities are Maternal Empathic Understanding (Oppenheim et al., 2001), “Reflective-functioning” [RF; elaborated by Fonagy et al. (1991a)], and Mental Orientation [MO or Mind-Mindedness by Meins (1997)]. Mind-Mindedness (MM) refers to the ability of the caregiver to form “appropriate representations of the child’s mind during real-time parent-infant interactions” (Yatziv et al., 2018b), whereas parental reflective functioning (PRF) refers to “the parents’ capacity to hold the child’s mental state in the mind” (Slade, 2005). This is referred to as parental mentalization, defined as the parent’s capacity to see their child as having a mental state of his/her own and to understand the mental state underlying behaviors in oneself and others (Luyten et al., 2017). The present paper aims to underline the possible effects of preterm birth and early parenthood on the elaboration of parental representations about the child.

A literature review was conducted to identify the extent to which parental representations can be a vulnerability factor in the context of preterm birth, and a challenge for early intervention programs. The aim of this article was to present an overview of the current findings on PR after premature birth. Here, we will focus on parents’ internal working models about their child and not the social representations of preterm birth.

We selected articles in the last two decades relating to parental (both paternal and maternal) representations after preterm birth. We used PubMed, Science Direct, and EBSCOhost as academic search engines, with the following terms in titles, abstracts, and main texts: “representations,” “perceptions,” “mentalization,” “reflective functioning,” and “parental,” or “mother,” “father,” “maternal,” and “preterm,” or “prematurity,” “low-birth weight,” or “premature”. This allowed us to identify 297 unique peer-reviewed articles published between January 2000 and October 2022, 30 of which we reviewed here. A woman’s self-image as a “mother” has been shown to be dependent on the woman’s attachment style (Zdolska-Wawrzkiewicz et al., 2019). We therefore focused on the parents’ representations of the child and excluded studies evaluating the adult’s past attachment quality. We reviewed studies conducted on parents of children born preterm. Moreover, we excluded articles that were not written in English or French and did not address parent-specific representations about the child, such as social representations, neonatal nursing staff perceptions, body representations, or child attachment representations.

Among the articles, we selected those that were related to the review’s topic: (1) the assessment and evaluation of the quality of PR in the context of preterm birth, (2) the link between PR and the attachment style of children born prematurely, (3) the influence and role of PR on the development of early parent-child interactions, and (4) the mechanisms underlying the construction of PR after preterm birth. It is important to note that this narrative review only highlights a portion of the literature as other related articles could be added by expanding search criteria. However, we believe that this review may help in the understanding of apparent contradictions and challenges involved in studying parental representations within the context of preterm birth.

First, we will present the comparative findings on the quality of PR after preterm birth and the assessment tools used in this specific birth context. Then, we will examine the link between PR and the attachment style of children born prematurely. Next, we will approach the influence of PR, on and through early interactions, according to the transactional model. We will then consider the role of parental constructs as either risk or protective factors before examining the influence of the mechanisms underlying the construction of PR. Finally, we will discuss clinical and research challenges faced when studying the role of PR after preterm birth in the context of a public policy centered on the child’s “First 1,000 days of life.”

## Exploring PR after preterm birth: assessment tools

The present review has allowed us to identify the different tools used or presented in the literature to assess parental representations about the child, two of which are specific to premature birth (Meyer et al., 1993; Bekhechi-Mistycki and Guédeney, 2008). While other tools exist to explore the parents’ own attachment representations [i.e., Adult Attachment Interview, AAI; by George et al. (1985)], the tools we chose to review aimed to capture the parent’s understanding and knowledge of the child based on parenting experiences and the parents’ representations of their relationship.

First, we identified the *Working Model of Child Interview* (WMCI) developed by Zeanah et al. (1994). This tool has been shown as being sufficiently valid and clinically useful (Vreeswijk et al., 2012) and has been mostly used in the studies we reviewed. The purpose of the WMCI is to explore the quality of parental attachment representations toward a particular child. Parenting quality is explored through the subjective experience reported by the parents (e.g., relational episodes with strong emotional content), which is specific to the dyadic relationship. In a structured interview, the content and affective tone of the discourse reflect the parents’ mental representations. The narrative style and emotions experienced by the parents are also examined. The analysis of the parents’ responses allows the investigators to identify different types of parental representations, namely, balanced, disengaged, and distorted. The parents with balanced representations express coherent and clear statements and give the impression of being at ease in their role and having a clear knowledge of their child’s needs and temperament. In the case of disengaged representations, the parents show a lack of interest in the child and express a distant relationship with poorly developed narratives. Finally, distorted representations are characterized by disconcerted descriptions and an overrated narrative, in which the parents try to highlight their involvement with the child. Borghini et al. (2016) have supplemented this tool with the *Reflective Function Scale* (Fonagy et al., 1991a) in order to assess the quality of maternal reflective functioning.

The *Parent Development Interview* [PDI-Revised by Slade et al. (2004)] is based on open-ended questions asking parents to describe the child, episodic memories of daily life with the child, the parent’s feelings about their role as a parent, and their reactions to the child’s behaviors. The questions are designed to capture the parents’ ability to elaborate and reflect on emotional experiences from the child’s perspective, as well as their own emotional experiences. Finally, the parents are asked to think about the similarities and differences that they could establish between their own behavior and that of their own parents. Thus, the PDI explores both the parents’ own early and past emotional experiences, their representations of the relationship with the child, and their parenting experiences. This tool not only aims to explore the parents’ ability to elaborate on the child’s behavior, thoughts, and emotions but also the parents’ capacity to access their own feelings linked to their parenthood.

The *R-Interview* is a questionnaire published and validated by Stern et al. (1989) but is not widely used in the studies we reviewed. Fifty-four questions divided into five topics aim to evaluate maternal representations of (1) her child, (2) her own mother, (3) herself as a mother, (4) herself as a woman, and (5) the child’s father. To this end, the mother is instructed to mark a 10-centimeter bipolar scale with pairs of opposing characteristics related to each topic. The scores are calculated as the measured distance (in millimeters) between the negative pole and the mark, resulting in a topic-specific score and an overall score of quality of maternal representations ranging from 0 (most negative representation) to 100 (most positive representation).

The *Clinical Interview for Parents of High-Risk Infants* [CLIP by Meyer et al. (1993)] is a semi-structured interview designed to assess eight main domains related to the child’s premature birth. These domains focus on the infant’s current status, memories related to the pregnancy, the experience of the delivery, the relationship with the baby, feelings as a parent, the parent’s reactions to the neonatal intensive care unit (NICU) environment and staff, the relationship with family and social support perceived during this time, and, finally, the hospital discharge and future (Meyer et al., 1993; Keren et al., 2003; Candelori et al., 2015). The CLIP specifically investigates maternal readiness or rejection, the mother’s coping skills, and her ability to make projections for the future in this specific birth context. Keren et al. (2003) developed a coding scheme based on the parents’ narrative style and the affective tone of the interview. In the Italian validation of the tool (Candelori et al., 2015), the CLIP has been used to assess both paternal and maternal representations after preterm birth, with a better adaptation of the coding used for maternal representations.

The last tool identified in the literature is the Bekhechi-Mistycki and Guédeney’s (2008) Questionnaire. It aims to evaluate the mother’s representations concerning her ability to focus on the present (i.e., reporting the child’s current needs and abilities), and to make projections and share concerns about herself regarding the future. The foundation of this semi-structured interview is the attachment theory, hence, allowing the exploration of the evolution of parental representations and attachment strategies used by the mother to cope with the traumatic nature of her child’s premature birth. These dimensions can be assessed through the content of the parent’s representations and narrative style, as well as taking into account the presence or absence of trauma resolution references in the parent’s narrative. This tool was designed to assess maternal representations specifically in the case of preterm birth, but it seems to be more of a tool for clinical investigation (e.g., to explore the mother’s state of mind and her ability to overcome the trauma following a premature birth) rather than for a standardized assessment.

The tools listed above have been used in the studies cited in this narrative review, with the exception of the questionnaire of Bekhechi-Mistycki and Guédeney (2008), in order to assess PR in the context of preterm birth.

## Quality of PR after preterm birth: research findings

Parents of infants admitted to the NICU may have negative perceptions of their premature baby. Despite reacting differently shortly after their child’s birth, both mothers and fathers experience intense fear of losing their child or the potential for long-term disabilities (Löhr et al., 2000). Yet, the influence of the child’s birth status (preterm vs. full-term) on the quality of parental mental representations has been mostly investigated in mothers. Notably, Korja et al. (2009) conducted a comparative assessment of maternal attachment representations in Finnish primiparous mothers of preterm (born before 32 GW) and full-term infants at 12 months of age. No significant differences regarding the distribution of the three main categories of representation (balanced, disengaged, and distorted) have been observed between the two groups. For instance, 55% of the mothers in the preterm group shared balanced representations while in the full-term group (versus 69% of the mothers in the full-term groupe). Nevertheless, qualitative differences have been found between the two groups: mothers of preterm infants scored lower on the Coherence, Acceptance, and Fear for Child Safety scales compared to mothers of full-term infants. These findings suggest that preterm birth of children can have disrupting effects on parental expectations. Similarly, Cox et al. (2000) have reported a high percentage of balanced maternal representations of the infant within their sample of mothers of high- and low-risk preterm infants. Hall et al. (2015) have found consistent results when investigating the proportion of disrupted representations between the three gestational age groups (very preterm, moderately preterm, and full-term) 6 months after the child’s birth. No significant difference was found between the three groups: 23.5% of mothers of full-term infants, 19.5% of mothers of moderately preterm infants, and 21.6% of mothers of very preterm infants had disrupted representations. In contrast, Forcada-Guex et al. (2011) have found that mothers of children born at full term (25 full term children) had more balanced representations of attachment with the infant compared to mothers of children born before 34 GW (47 preterm children), assessed at 6 months of corrected age (CA). Altogether, these findings suggest that prematurity does not necessarily generate disrupted nor non-balanced maternal representations of the preterm child.

A longitudinal study conducted by Fuertes et al. (2011) has shown that mothers who gave birth prematurely expressed more negative feelings about their pregnancy and delivery and showed concerns about their child’s future health and development. Although no significant difference was found between mothers of full-term and preterm children regarding maternal representations of the 9-month-old child’s temperament, preterm infants were perceived as more difficult to soothe with a pacifier and to dress and undress. However, mothers of preterm infants who had expressed more positive feelings at birth about their child’s future development perceived their 9-month-old child as less difficult.

While the majority of studies focused on assessing maternal representations in the context of preterm birth, Tooten et al. (2014) were the first to explore both maternal and paternal representations after very premature birth (occurring before 32 GW), moderate premature birth (occurring between 32 and 37 GW), and full-term birth. In this study, no significant association between the distribution of attachment representations and the child’s prematurity or the parents’ gender was found. However, the authors did find a significant difference between the gender of the parent in the non-balanced representations group: more distorted representations were assessed in mothers who reported being confused about their role whereas fathers showed more disengaged representations, characterized by withdrawal behavior. Taken together, these studies have raised the question of risk factors leading to the development of unbalanced parental representations after a child’s premature birth. Based on our literature review, we have identified two risk factors that influence the quality of PR: the degree of prematurity and parental stress.

### Influence of the degree of prematurity on the quality of PR

As seen above, preterm birth is a specific birth context that may lead to distorted or disengaged representations. For example, only 20% of mothers of a 6-month-old child born prematurely (30% at 18 months) had secure attachment representations, compared to 53% of mothers of children born at term (57% at 18 months) (Borghini et al., 2006). However, when investigating the effects of preterm birth on PR, the degree of prematurity should be considered. Tooten et al. (2014) revealed that timing of preterm childbirth (i.e., moderate or very preterm birth) did not affect the quality of attachment representations. Yet, mothers of moderately preterm children (born after 32 GW) perceived more positively their infant and their relationship (as assessed by their scores on CLIP) compared to mothers of very preterm infants (born before 32 GW) who experienced a greater fear of loss (Trumello et al., 2018). This anxiety concerning the child’s safety persisted among parents of very preterm infants six months after childbirth (Tooten et al., 2014). Indeed, given the degree of prematurity, the newborn could be exposed to a high medical risk, which could, in turn, affect the parental/NICU experience. For instance, mothers of high-risk infants did not feel prepared for motherhood compared to mothers of low-risk children (Keren et al., 2003). When assessing PR in a group of mothers of preterm children, the mothers of premature children with low medical risk showed more disengaged representations, whereas the mothers of premature children born with high medical risk expressed more distorted representations (Borghini et al., 2006). These results suggest that the severity of the child’s perinatal risk could influence the type of maternal attachment representations. Overall, despite the older gestational age of the preterm child and a lower risk of medical complications, parental representations appear to be impacted by the child’s premature birth.

Maternal experience reported from the first hours of the newborn’s life provides a better understanding of the influence of the child’s preterm birth, especially the degree of prematurity on the maternal reaction in the postnatal period (Gonçalves et al., 2020). Mothers of preterm infants (born between 32 and 36 GW) expressed fear following the unexpected occurrence of the child’s preterm birth and the associated risks. However, they remained optimistic about their ability to care for their baby, while mothers of very preterm infants (born before 32 GW) already expressed more negative feelings, such as distress, and showed difficulties in their ability to care for their child. On the other hand, mothers of full-term infants presented their babies as calm more often and did not expect difficulties in taking care of them. An increased risk to the child’s health exacerbates the emotional distress parents may feel and have to cope with. They remain “past-oriented” when presenting the child’s behavior (using the CLIP) at 3 months of CA (Cataudella et al., 2016). It is difficult for parents to create and maintain a more positive representation of their prematurely born child when the child’s future is uncertain, as this triggers parental perception of the child’s vulnerability (Tallandini et al., 2015).

However, it should be noted that at 18 months of age, a greater proportion of balanced maternal representations are expressed about the toddler (Borghini et al., 2006; Meijssen et al., 2011). This suggests that the parents become more confident about the child’s future afterward, leading to more balanced parental representations. When taking into account the impact of the degree of prematurity and the severity of the child’s medical risk, one could also question the influence of stress on PR.

### Influence of parental stress on PR after preterm birth

Premature birth is often perceived as a stressful context for the parents. Yet, premature birth in itself cannot be considered as the only source of stress. The actual trauma associated with this complex situation is more related to the way in which this mostly unexpected event is experienced and apprehended by the parents. According to Bekhechi-Mistycki and Guédeney (2008), the parents who remain focused on the past are still seeking justification for the event and, thus, retain the traumatic nature of the premature birth experience. Experiencing a child’s preterm birth has a negative impact on the parents’ feelings and perceptions who then experience difficulty in focusing on the current reality and attending to the child’s actual needs and emotions. Bekhechi-Mistycki and Guédeney (2008) have suggested that the resolution of the traumatic experience lived by the parents is favored by the parents’ ability to focus on the present reality and to share a realistic vision of the vulnerable child’s conditions and abilities. Analysis of the parents’ discourse when using the CLIP also reflects the traumatic nature of having a child born preterm (Forcada-Guex et al., 2009). Indeed, the birth is presented by the parents as a moment of shock and a source of confusion between the expected child and the actual newborn child. Consequently, distorted or disengaged representations can emerge from parental anxiety, thereby impacting parental traumatic reactions after the child’s premature birth (Borghini and Muller-Nix, 2015). For instance, mothers with distorted representations often emphasize their own emotions over their relationship with their preterm child, resulting in higher PTSD symptoms. Conversely, mothers with disengaged representations tend to experience emotional numbing and report fewer PTSD symptoms (Borghini and Muller-Nix, 2015). This has been noted by Forcada-Guex et al. (2011) in mothers with high post-traumatic stress scores who expressed more distorted representations, whereas mothers with low post-traumatic stress scores expressed more disengaged representations. This evidence provides insights into the previously mentioned findings by Borghini et al. (2006), suggesting that both the child’s high perinatal risk and greater parental distress could induce distorted representations. Furthermore, maternal depression symptoms have been shown to be associated with non-balanced representations (Keren et al., 2003; Korja et al., 2009; Trumello et al., 2018). Trumello et al. (2018) found that the scores on the Edinburgh Postnatal Depression Scale [EPDS by Cox et al. (1987)] significantly predicted the mothers’ self-image, perceived support and readiness for hospital discharge in the CLIP. Parental experience and reactions after a preterm delivery have also been shown to differ between fathers and mothers (Löhr et al., 2000; Gamba Szijarto et al., 2009; Stefana et al., 2022) and among preterm fathers (Stefana et al., 2018). This difference between the parents’ coping strategies may explain the non-systematic effect of preterm birth on the quality of parental representations found in the literature. It may also account for better representativeness in the CLIP scoring for maternal rather than paternal representations (Candelori et al., 2015).

Given the complexity of the processes involved, prematurity appears to be a context of vulnerability that is likely to impact the development of balanced parental representations about the child and optimal early affective parent-infant interactions. In this regard, the potential link between PR and attachment quality in children born preterm has been explored.

## The role of PR on the attachment quality of the preterm child

Previous studies focusing on the factors impacting the quality of PR after preterm birth have shed light on the need to take into account the possible role of PR on the attachment quality of the preterm child. For instance, Cox et al. (2000) have shown that maternal attachment representations of the infant, but not the mother’s infantile history (referring to her past attachment representations), significantly predicted infant attachment security for their sample. These findings point toward a more important role of maternal factors rather than the infant’s neonatal status in determining infant attachment style. In support of this claim, Laganière et al. (2003) found a mediating role of maternal perceptions (assessed by Abidin’s Parenting Stress Index) on the quality of mother-infant attachment security when the child is born preterm. For example, when the mother perceives her child more negatively, she’s more likely to develop a more insecure attachment relationship with her preterm infant. However, preterm children with neurological impairments may be more at risk to develop disorganized attachment patterns (Cox et al., 2000).

Abergel and Blicharski (2013) assessed attachment styles using the Q-SET among 38 preterm and 38 full-term children aged between 22 and 24 months, whose mothers were asked to report their child’s perceived attachment behaviors. The results did not reveal a significant proportion of insecure attachment styles in children born prematurely. Yet, for preterm children with a secure attachment style, hypervigilance was still reported in the mothers. According to these findings, the hypothesis of a maternal compensatory behavior linked to the child’s preterm birth could be advanced. Using the Strange Situation Procedure, Fuertes et al. (2011) have found an association between maternal representations of the child’s difficult temperament at 9 months of age and infant attachment style at 12 months of age in preterm infants. For example, preterm children who were introduced by their mother as having an easier temperament at 9 months were those assessed as having a secure attachment style. Taken together, these studies seem to be consistent with previous findings, indicating no prevalence of insecure attachment in preterm children. Instead, the attachment style assessed in preterm children appears to be associated with maternal representations of the child’s temperament or the child’s attachment behaviors. Moreover, the hypervigilance behavior evidenced by Abergel and Blicharski (2013) in mothers of securely attached preterm children raises concern about early interaction quality. Consequently, the link between PR and interactive dynamics in parent-preterm infant dyads has been investigated.

## The influence of PR on specific dyadic interaction patterns

Several studies have examined the impact of preterm status on mother-infant interactions and, more precisely, the link between the type of maternal representations and specific dyadic patterns. For instance, Stern et al. (2000) demonstrated the impact of preterm birth status (called the prematurity stereotype) on the mother’s interactive behavior by introducing full-term children as premature to the mothers. The observational findings revealed that despite interacting with a full-term baby, the mother touched him or her less. Hence, children born prematurely seem to be exposed to an exaggerated perception of persistent vulnerability which impacts the mother’s engagement during early interactions. When assessing mother-preterm infant dyadic interactions, Forcada-Guex et al. (2011) identified two possible dyadic interaction patterns that emerge significantly: a controlling and a cooperative pattern. The cooperative interactive pattern is characterized by a more sensitive maternal behavior and optimal dyadic interactions, and it promotes a secure attachment style in the child. On the other hand, a controlling dyad is characterized by maternal controllingness and a compulsive-compliant behavior pattern in the child during the dyadic interaction. Dyads that were characterized as neither cooperative nor controlling (e.g., maternal unresponsive behaviors) constituted the heterogeneous dyads.

In addition, Forcada-Guex et al. (2011) reported a link between the interactive dynamics of mother-preterm infant dyads (born before 34 GW) assessed at 6 months corrected age, and maternal attachment representations of their child. Cooperative dyads and balanced representations have been more often found among mothers whose children were born at term compared to all mother-preterm infant dyads. In contrast, mothers of preterm children who were engaged in heterogeneous and cooperative dyads shared more distorted representations compared to mothers of full-term infants. Mothers of preterm children who displayed controlling behaviors when interacting with their children also expressed more disengaged representations (Forcada-Guex et al., 2011). The impact of negative parental perceptions on parent-child exchanges extends beyond the NICU stay and continues upon the return home. Moreover, permanent parental preoccupations with the premature newborn’s health can disrupt early interactions. Porter et al. (2009) found that mothers of preterm infants who perceived other infants labeled as “preterm” more positively at 5 months also exhibited more positive interactions with their infants at 9 months. These results suggest an association between perceived vulnerability and the mothers’ later interactive behavior within the context of preterm birth.

Interactive dynamics of the mother-infant dyad offer an overview of the context of cumulative vulnerabilities associated with preterm birth. Maternal representations influence the child’s attachment style through early maternal interactive behavior (assessed at 6 months postpartum), whether with children born at term or prematurely (Hall et al., 2015). *Hall et al. (2015) also found that maternal disrupted representations predicted insensitive and intrusive maternal behavior during interactions with toddlers aged 6 and 24 months*. Mothers of moderately preterm children displayed more sensitivity than mothers of very preterm infants, but no other differences were found when comparing maternal interactive behavior among mothers of full-term, moderate, and very preterm infants (Hall et al., 2015). However, it should be noted that the link between distorted parental representations and greater at-risk dyadic interaction contexts has been evidenced for dyads with both children born at term and preterm (Korja et al., 2010). More positive representations in the mothers’ narratives about their preterm infant and their experience of motherhood (as assessed by the CLIP) have been shown to support mother-infant bonding through affective maternal touch (Keren et al., 2003). On the other hand, maternal negative perceptions of the baby (e.g., contradictory projections for the baby’s future) have been shown to predict non-optimal mother-infant interaction (i.e., infant withdrawal behavior) (Keren et al., 2003).

The studies reviewed above have suggested that maternal representations were associated with specific dyadic interactive patterns, particularly disruptive ones. However, Forcada-Guex et al. (2009) observed that only 23% of the mothers of preterm children in cooperative dyads shared balanced representations. These surprising results raise the question of the protective factors at work within cooperative dyads that did not express balanced representations. Parental representations can be enriched by the dyad’s subjective experience. As a result, parental sensitivity to child cues and more optimal dyadic interactions may support the construction of more balanced representations.

## PR resulting from the co-construction of parent-child interaction

Parental representations of the child consist of the exploration of current and past expectations and fears experienced by the parent but also tend to integrate elements of the actual parent-child relationship, particularly through the care provided by the parent to the child (Bekhechi-Mistycki and Guédeney, 2008). In this regard, the child contributes to the dynamics of the parent-child relationship under construction and is part of a system of intersubjective representations.

As presented earlier, the experience of premature birth can generate exaggerated anxious concerns in the parent, who will be engaged in more frequent intrusive behaviors, or conversely, withdrawal behaviors translated by the avoidance and minimization of actual difficulties (Keren et al., 2003; Hall et al., 2015; Trumello et al., 2018). Disturbed parental representations constitute a risk factor for non-optimal early affective interactions and parent-infant future attachment bonds. The anxiety generated by this traumatic event can fix the image of a vulnerable and irritable child in the parents’ minds. On the other hand, the parents can fear a major developmental delay and over-stimulate their child. Therefore, PR can influence the dynamics of early parent-child interactions through the parents’ behavior toward the child (Keren et al., 2003; Forcada-Guex et al., 2011; Hall et al., 2015) which in turn influences the child’s interactive engagement (Trumello et al., 2018). Defensive (e.g., emotional disengagement) or distorted parental perceptions of the child may constitute a difficulty for the parents to adjust their behaviors to infant cues, leading to low levels of parental sensitivity toward the child (Hall et al., 2015). However, PR cannot be dissociated from the construction of early parent-child interactions. Indeed, PR is part of parent-child interactions based on dyadic reciprocity (e.g., synchronous temporal contingencies). In some cases, impervious parental representations can lead to the parents denying the child’s actual abilities, thus impacting the latter’s interactive engagement with the parents. Subsequently, the poor interactive engagement of the child will reinforce the distorted perceptions shared by the parents about the child’s temperament or interactive style. Keren et al. (2003) have shown that high-risk infants were touched less often by their mothers who expressed negative attitudes toward motherhood and were also more withdrawn during mother-infant interaction (Keren et al., 2003). Conversely, mothers who reported more positive representations of their preterm infants and their experience in NICU elicited their infant’s interactive behavior (i.e., through tactile contacts) and had more adaptive responses to their infant cues (Keren et al., 2003). It is therefore necessary to identify how factors associated with preterm birth can favor impervious or instead, flexible PR.

## Roles of parental mental elaboration mechanisms for parenting after preterm birth

According to Slade (2005), PR can be apprehended through the parents’ narrative and ability to interpret their own behaviors and those of the child resulting from mental states such as emotions, desires, and intentions (referred to as paternal and maternal Reflective Functioning, RF). The parent’s ability to organize and make sense of parenting has thus been explored through parental mentalization, which encompasses both PRF and MM.

Studies investigating the influence of preterm birth on the quality of PR have identified cumulative vulnerability factors. Recent research is now focusing on the role of specific PR elaboration mechanisms in the context of preterm birth. Borghini et al. (2016) have explored mentalization through attachment interviews conducted with mothers and children (full-term and preterm) 18 months and then 11 years after birth. They have shown that the quality of maternal mentalization was affected by preterm birth, especially 6 months after the child’s birth. In contrast to the group of mothers of moderate-risk preterm infants, maternal RF abilities (assessed 18 months after birth) were significantly lower in the group of mothers of high-risk preterm infants compared to the control group. Moreover, there was a significant correlation between the mothers’ RF scores at 6 and 18 months and the child’s mentalization scores at 11 years old. However, the transmission of maternal RF abilities to the child was not found in the group of high-risk preterm infants whose mothers had high mentalization scores. Borghini et al. (2016) have thus suggested that maternal mentalization can be impacted by parenting stress experienced in preterm birth and can also influence the emotional regulation abilities of the preterm child.

Maternal mentalization contributes to the mother’s sensitive response during mother-infant interaction (Yatziv et al., 2018a). However, this role becomes weaker in highly stressful contexts with cumulative stressors, such as mothers who experienced preterm birth in a chaotic home environment. Consistent with this, Suttora et al. (2020) have shown that among mothers of preterm children, those who reported higher levels of parenting stress also faced greater difficulties in accurately describing their child’s mental states during mother-infant interaction. This was observed despite the mothers of preterm infants having similar levels of parenting stress and MM compared to mothers of full-term infants (Suttora et al., 2020, 2021). Indeed, preterm birth, as a stressful context, interferes with the associations between maternal executive functions (EFs) and maternal mentalization (i.e., appropriate MM) (Yatziv et al., 2018b). Although EFs support the elaboration of maternal mentalization in specific challenging contexts (e.g., perceiving the child as difficult or experiencing co-parenting dissatisfaction), preterm birth instead tends to “elicit automaticity” (Yatziv et al., 2020). Experiencing preterm birth creates a situation of cumulative vulnerabilities affecting mothers’ abilities to mentalize about their child. This, in turn, impacts maternal sensitivity during dyadic interaction. However, Suttora et al. (2021) have found that in their sample of both mothers of preterm and full-term infants, maternal MM was not directly impacted by the traumatic experience of the child’s preterm birth nor parenting stress. The authors have instead suggested that perceived social support’s contribution to maternal MM depends on the levels of parenting stress experienced by the mothers. Indeed, Suttora et al. (2021) have evidenced that among mothers who felt emotionally supported, only those who experienced low to mild parenting stress showed improved MM capacities.

In a recent study conducted by Ruiz et al. (2020) on parents’ capacity to mentalize (i.e., RF) and parenting-related concerns (assessed by the PDI), RF scores did not differ between parents of preterm or children born at term, which has also been reported by Dollberg et al. (2022). However, among parents of preterm children, fathers showed lower RF scores compared to mothers (Ruiz et al., 2020). The study has also revealed qualitative differences between fathers and mothers in their parenting concerns and attitudes related to their RF scores. To be more specific, mothers of children born preterm were especially focused on their ability to understand their child’s thoughts and emotions, while fathers pondered more about successfully fulfilling their role as a father. However, as indicated by Ruiz et al. (2020), the generalizability of the results may be restricted, despite RF offering the potential for valuable insights for studying parenting.

Similarly, Dollberg et al. (2022) pointed out the importance of considering both mothers’ and fathers’ RF to provide support for parenting. They found that the parents’ self-focused RF moderated the link between preterm birth and parenting perceived stress, in different stress domains. Among families with a premature infant, parents experienced less personal distress when the mothers demonstrated a better capacity for self-reflection, while greater stress in parenting the child was reported when fathers exhibited higher levels of focus on their own mental state. Taken together, these findings raise the importance of examining the role of parental mentalization in specific contexts and investigating the influence of PRF on family triadic interaction, particularly in the case of preterm birth.

It is noteworthy that recent studies have included both fathers and mothers when investigating the influence of preterm birth on mental elaboration mechanisms among parents of preterm children. Taken together, the studies reviewed above highlight the importance of assessing both the fathers’ and mothers’ parenting concerns in the context of preterm birth and providing support specific to each parent’s needs. Parental elaboration mechanisms (notably PRF) also play a key role in the parenting experience, especially as a moderator in the link between preterm birth and parenting stress (Dollberg et al., 2022).

## Discussion

In this review, we discussed the singularities and the role of PR following preterm birth regarding the dynamics of early parent-infant interactions. Studies investigating the social representations of prematurity among caregivers or children born prematurely are not addressed in this review.

After reviewing the aforementioned studies, we were able to establish that: (1) most of the studies we reviewed assessed maternal representations after preterm birth with a notable lack of data on paternal representations; (2) research studies did not find a direct link between preterm birth and the quality of PR but rather address the various risk factors associated with preterm birth which may be linked to distorted or disengaged PR following a child’s preterm birth; (3) assessing maternal representations provide insight into the future development of the mother-infant relationship and specific dyadic patterns; (4) early interactions are also part of the co-construction of PR which in turn can influence maternal and child interactive behaviors; (5) recent research has examined the mechanisms underlying the construction of PR in the case of preterm birth, which has been shown to be impacted by stressful contexts (such as preterm birth) and parental gender. Those mechanisms could then constitute long-term vulnerability factors for the parent-preterm dyad (e.g., parental perceived stress).

### Clinical implications

Additionally, findings stress the need for the early identification of vulnerable dyads by identifying markers found in parental narratives in order to provide targeted interventions on both parents’ experience of preterm birth. Parents’ experience of prematurity reveals that fathers and mothers react differently (Ionio et al., 2016): fathers are highly sensitive to the severity of prematurity, as severe cases tend to trigger PTSD symptoms (Gamba Szijarto et al., 2009). In contrast, mothers tend to develop PTSD symptoms in response to prematurity (Gamba Szijarto et al., 2009). PTSD symptoms have been shown to elicit distorted representations of the child (Forcada-Guex et al., 2011). Therefore, intervention programs should provide specific support to each parent, as evidenced when comparing the experiences of fathers and mothers of severe preterm children (Provenzi et al., 2016). Strengthening the parent-child relationship while focusing on parenting skills thus contributes to resolving the traumatic experience, elaborating balanced PR, and improving the socio-emotional development of preterm infants.

This review also emphasizes the complexity and multiplicity of factors influencing child development. Consequently, no direct causal link between the child’s premature birth and the risk of insecure attachment was evidenced, which constitutes a reassuring and destigmatizing message for professionals and parents.

### Theoretical implications and perspectives for future research

On the other hand, preterm birth remains a source of vulnerabilities regarding the child’s developmental outcomes (Forcada-Guex et al., 2009). Multiple factors such as parental emotional distress may lead to more rigid parental representations impervious to the child’s actual abilities and characteristics observed during parent-child interactions (Borghini and Muller-Nix, 2015). Indeed, PR, particularly maternal representations, could act as “mental filters” and interactive filters. In the case of disturbed representations, specific interaction patterns amplify vulnerabilities of parent-infant dyads, illustrating the observable cases of “over-disability” (Bullinger, 2007) and the pivotal role of early experiences in infant development (Sroufe et al., 2010).

In light of the aforementioned results and issues, it seems relevant to question the modalities by which the role played by PR in early parent-preterm infant interactions could be investigated to identify specific interactive models. The influence of the degree of prematurity on the quality of maternal representations identified by Borghini et al. (2006) would likely be related to better emotional access to mothers of children with a more severe degree of prematurity during the NICU stay. In contrast, mothers of moderately preterm children would rather tend to avoid expressing their feelings (Borghini and Muller-Nix, 2015). If parents’ emotional access is determined by contextual factors and setting (staff support and length of the NICU stay) around which early interactions are experienced during the hospital stay, this would illustrate the co-construction model of PR through parent-child interactions.

For future research, it would be pertinent to rely on the assessment of PR after a premature birth in order to highlight the extent to which this “mediating variable” could serve as a protective or vulnerable factor regarding parent-child attachment bonds and the children’s outcomes. This could be achieved by exploring the interactive dynamics of the parent-child dyad. The longitudinal study conducted by Costantini et al. (2017) revealed that maternal MM plays a strong role in preterm infants’ expressive language development. Further studies examining the influence of parental mentalization on the sensory and affective modalities used by the parent, particularly during developmental care, could allow for a better understanding of PR’s influence after preterm birth through specific interaction models. These potential findings would support the postulation of the co-construction of PR through dyadic interactions, as suggested by the transactional model proposed by Sameroff (2009).

The parent-infant dyad is organized as “an intersubjective meeting space that expands during the first year of life” (Rochat, 2006, p.133). Early interactions allow the child to learn about others and himself/herself (Aubineau et al., 2017) while being sensitive to vulnerability factors. PR is the key transmitter of parental vulnerability in the case of traumatic birth. Future studies may then question the impact of disrupted PR as “filters” in the development of social cognition among preterm infants. In fact, young people born prematurely reported their parent’s overprotective behavior (Leavy et al., 2015).

In addition, findings regarding the influence of maternal mentalization abilities on the abilities of the previously premature 11-year-old child (Borghini et al., 2016), highlight the importance of studying the mechanisms of PR construction in order to prevent socio-emotional and developmental difficulties. However, there is a lack of consensus regarding the mechanisms of PR construction and their organization. Therefore, the need to develop a model presenting the different levels of mental representations specific to the parental function, as already pointed out by Demers et al. (2009), is underlined. Moreover, few studies have explored both maternal and paternal representations, with the majority focusing on mother-preterm infant interactions. Yet, the fathers’ role in the child’s development is complementary to the mothers’ involvement (Miljkovitch and Pierrehumbert, 2005). The recent systematic review by Larsson et al. (2022) points out the importance of considering the triad after a child’s premature birth. The systematic review conducted by Charpentier Mora et al. (2023) confirmed the influence of paternal mentalizing on both the fathers’ parenting features and the child’s outcomes. It is therefore crucial to investigate the role played by paternal representations, particularly fathers’ mentalizing specific influence, in the context of preterm birth. As commented by Taubner (2020), it would be beneficial to examine if fathers’ higher mentalizing could “counterbalance” lower mentalizing in the mother in the context of preterm birth. The tools listed in this review stress the need to create a clinical and research tool specific to prematurity, evaluating both parents’ representations, and thus providing a reliable assessment of the quality of PR in the case of preterm birth.

We encourage future studies to investigate these research questions from a developmental perspective. Indeed, the attachment development of children born preterm is not solely the consequence of maternal interactive behavior (e.g., maternal sensitivity) (Miljkovitch et al., 2013). For instance, Wolke et al. (2013) conducted a study that identified disorganized attachment patterns in very preterm infants. However, considering context-specific factors can offer valuable insights into these results and ultimately, the attachment development of preterm infants. Moreover, screening for unbalanced prenatal representations is predictive of less optimal social-emotional functioning in young 24 months-old children born at term (Guyon-Harris et al., 2021), as well as predicts the quality of the parent-child relationship (Vismara et al., 2022). This would allow, specifically in the context of preterm birth, the referral of vulnerable dyads to early intervention programs. Indeed, an early intervention program has already been offered to mothers of children born before 32 GW between birth and 6–8 months of age to study the effect of these interventions on the quality of maternal attachment representations assessed at 18 months (Meijssen et al., 2011). However, the lack of data collected on the quality of PR at birth does not allow us to assess the contribution of this intervention to PR in the case of preterm birth. A follow-up would also allow for a better understanding of the different factors related to the potential impact of premature birth on prenatal representations and a better grasp of assessed factors in the postpartum period (Yatziv et al., 2020). Research findings could be shared with neonatal care teams and services to promote early prevention intervention programs, such as prenatal interviews or parental guidance, during the NICU stay and when returning home.

## Conclusion

To conclude, it is of high importance to not only focus on considering the causal pathways between PR and attachment style but also prioritize examining the precursors of the construction of intersubjective bonds and the parent-preterm infant interactive dynamic. The study of both elements will determine protective and risk factors regarding the transmission of parental vulnerability during the child’s early development. Further studies aimed at exploring paternal representations; especially, the role played by fathers’ mentalizing in the case of preterm birth should be promoted. These are the new challenges of early prevention embedded in the policy of the “First 1,000 days.”

## Author contributions

All authors contributed to the manuscript revision, read, and approved the submitted version.
